# The presumed oldest flying insect: more likely a myriapod?

**DOI:** 10.7717/peerj.3402

**Published:** 2017-05-30

**Authors:** Carolin Haug, Joachim T. Haug

**Affiliations:** Department of Biology, Ludwig-Maximilians-Universität München, Planegg-Martinsried, Germany

**Keywords:** Chilopoda, Rhyniognatha hirsti, Rhynie chert, Crussolum, Devonian insect

## Abstract

The early fossil record of insects is scarce, with only few finds in the Devonian. All these finds appear problematic and controversial, partly due to incomplete preservation and challenging interpretation of many structures. We provide details of one of these important forms, *Rhyniognatha hirsti* from the famous Rhynie Chert Lagerstätte with up-to-date 3D imaging techniques. The fossil has been interpreted as the remains of one of the earliest flying insects. The specimen mainly preserves the remains of the head. The structures of the mandibles have been used as a main argument for an interpretation as an insect, but these are in fact less easy to interpret. New observed structures include the remains of a head capsule and an additional pair of mouth parts. Structures formerly suggested to represent remains of the head capsule or apodemes are more likely to be representing glands of ectodermal origin. The newly observed structures do not support an interpretation as an insect. Instead they make the interpretation as a myriapod more likely, possibly as a centipede. Centipede remains from the Rhynie Chert are known from scutigeromorphs. We therefore point out that *R. hirsti* could be interpreted as an early centipede.

## Introduction

The group Insecta has been repeatedly supposed to represent a very diverse and successful group of animals (e.g., Fayers & Trewin, 2005; Rainford et al., 2014; see discussion in Haug, Haug & Garwood, 2016). Yet the origins of Insecta (in the sense of ‘Hexapoda’ in Anglo-American literature) are still largely unknown. Phylogenetic analyses of the last decades have produced a huge variety of presumed sister groups for Insecta (see e.g., discussion in Haug, Maas & Waloszek, 2010, their fig. 2; Rasnitsyn & Quicke, 2002; Newman, 2005; Glenner et al., 2006; Strausfeld & Andrew, 2011; Misof et al., 2014; von Reumont & Wägele, 2014).

The uncertainties of the early origins of Insecta is also reflected by the quite incomplete early fossil record of the group. The first fossils that should represent insects have been found in Devonian rocks, being as old as 400 million years. Yet most of these fossils are problematic at best, and still leave much to desire. We aim at providing a short overview of the difficulties of these (presumed) Devonian insect fossils before treating one of them in more detail.

### Presumed Devonian insect fossils

(1) Historically, the oldest record of Devonian insects is *Rhyniella praecursor*, a spring tail (collembolan) that was described based on material from the Lower Devonian Rhynie Chert Lagerstätte (Hirst & Maulik, 1926; Scourfield, 1940a; Scourfield, 1940b; Whalley & Jarzembowski, 1981). The specimens are exceptionally preserved and give access to minute details. Probably because of these fine details, the Devonian origin of the specimens has been questioned, suggesting them to represent extant contamination (Crowson, 1985). Yet this idea has been subsequently rejected (Greenslade, 1988), and *R. praecursor* is now largely accepted as a true 400 million years old fossil.

(2) The single specimen of *Rhyniognatha hirsti* was among the material originally described as *Rhyniella praecursor* but recognized as a separate species by Tillyard (1928). The specimen is fragmentary, mainly preserving parts of the head, most notably the mandibles. The morphology of these led Engel & Grimaldi (2004) and Grimaldi & Engel (2005) to interpret this specimen as a possible flying insect. Subsequently, *R. hirsti* has commonly been used to calibrate molecular clocks (e.g., Warnock, Yang & Donoghue, 2011; Misof et al., 2014; Wolfe et al., 2016) or to suggest that insects have first appeared in the Silurian (e.g., Grimaldi, 2010; Nel et al., 2013; Tong, Lo & Ho, 2016). Yet other authors have expressed doubts about the insect affinities of the specimen (e.g., Rasnitsyn & Quicke, 2002) and suggested that it could also represent a myriapod.

(3) *Eopterum devonicum* and *Eopteridium striatum* were originally interpreted as isolated insect wings from the Devonian of Russia respectively Ukraine (Rohdendorf, 1961; Rohdendorf, 1970). Based on their age they were originally thought to represent the oldest record of winged insects. Reinvestigations led to a re-interpretation as partially preserved tail fans of a malacostracan, possibly of mantis shrimp relatives (Schram, 1980).

(4) Historically, the next report of a Devonian insect fossil (Labandeira, Beall & Hueber, 1988) is a fragmentarily preserved archaeognathan (jumping bristletail) from Gaspé, Quebec. Comparable to *Rhyniella praecursor* the specimen has also been interpreted as an extant contaminant (Jeram, Selden & Edwards, 1990). Yet these assumptions were not based on re-investigations of the original material.

(5) The 380 million years old Gilboa material is renowned for exceptionally preserved cuticles of arthropods such as representatives of Chilopoda, Acari, Ricinulei and Trigonotarbida (Shear et al., 1984). Some fragments have been discussed as possible remains of insects as well. Among these possible remains are the cuticles of eyes of supposed archaeognathan affinities (Shear et al., 1984). Grimaldi & Engel (2005) additionally mention scales of possible archaeognathan or zygentoman affinities. While only representing fragments, these interpretations are plausible, but should be further supported by new investigations.

(6) *Devonohexapodus bocksbergensis* is the next fossil from the Devonian (Hunsrück shale, Germany) to be mentioned, although it was not interpreted as an insect, but as an early representative of the lineage towards insects (Haas, Waloszek & Hartenberger, 2003). It was thought to still retain many plesiomorphic features resembling also certain aspects of Myriapoda (supposed sistergroup, or even paraphyletic assemblage in the evolutionary lineage towards Hexapoda, according to the original authors). Together with *D. bocksbergensis*, two other species from the same Lagerstätte, *Wingertshellicus backesi* and *Cambronatus brasseli*, were also interpreted as possible derivatives of the early evolutionary lineage towards Insecta. The interpretation of *D. bocksbergensis* has been heavily criticized by various authors (e.g., Willmann, 2005), yet also accepted by others (e.g., Grimaldi & Engel, 2005). Therefore, although the species has been described relatively recently it has a ample history of interpretations (see Haas, 2005; Kühl & Rust, 2009 and references therein).

Similarities of *Devonohexapodus bocksbergensis* and *Wingertshellicus backesi* were already pointed out by Haas, Waloszek & Hartenberger (2003), but as the authors had no access to the material of *W. backesi* they could not draw further conclusions. The similarities between the two species led Kühl & Rust (2009) to synonymize the two species, rendering *D. bocksbergensis* as a junior synonym. The authors furthermore re-interpreted the general organisation of the body, pointing out the differences to insects, and in consequence excluding *W. backesi* as well as *C. brasseli* from the insect lineage.

(7) Another presumed insect fossil from the Devonian is *Leverhulmia mariae*, again from the Rhynie Chert (Fayers & Trewin, 2005). It was at first described as a myriapod (Anderson & Trewin, 2003), but later re-interpreted as a possible archaeognathan or zygentoman (Fayers & Trewin, 2005). The fact that *L. mariae* was at first identified as a myriapod and additionally cannot be ascribed with certainty to either Archaeognatha or Zygentoma demonstrates that also this fossil is not easy to interpret. The insect interpretation of *L. mariae* has not been questioned yet.

(8) *Strudiella devonica* is the latest addition to the presumed Devonian record of insects (Garrouste et al., 2012). Yet its very incomplete preservation makes its interpretation problematic, as for other presumed Devonian insects. The fossil has alternatively been interpreted as a decayed non-insect arthropod (Hörnschemeyer et al., 2013, but see also Garrouste et al., 2013).

All these examples show that the early fossil record of insects is not only scarce but in many cases problematic and hotly debated. Here we provide new information on one of these cases, *Rhyniognatha hirsti*. We have applied up-to-date imaging methods to the single specimen teasing out even smallest details that will shed new light on the systematic interpretation of this important fossil.

## Material and Methods

### Material

The single known specimen of *Rhyniognatha hirsti* was investigated. It is housed at the Natural History Museum London under repository number In38234. For comparison the head of a single specimen of the centipede *Scutigera coleoptrata* was documented, which came from the former teaching collection of the University of Ulm (Funke collection). Images will be deposited in https://www.morphdbase.de/ under the repository numbers C_Haug_20170507-M-10.1 to C_Haug_20170507-M-16.1.

### Methods

The specimen was documented on an upright Nikon Eclipse microscope with transmitted light. Objective lenses of 10×, 20×and 40×magnifications were used resulting in overall magnifications of 100×, 200× and 400×. Due to the specific optical properties of the chert resulting in a very limited depth of field, the specimen was documented with stacks, in higher magnifications with two adjacent stacks. Classical image fusion of these stacks does not yield sufficient results. We used the techniques described in Haug et al. (2009) and Haug et al. (2012) for projecting the stacks in 3D. Stereo images were used to present these projections. The stack could be turned 180° to allow a view on the other side (factually simple depth inversion). Interpreted structures are presented as color markings alongside the original projection. Based on these, simplified 3D models were produced in Blender.

The modern specimen of *S. coleoptrata* was documented on a Nikon Eclipse microscope exploiting the autofluorescence of the specimen. Workflow followed Haug et al. (2011). Mouth parts were sequentially removed to reveal the arrangement of underlying ones.

### Terminology

We try to apply a neutral euarthropod terminology, especially concerning the appendages (Haug et al., 2013). This seems not very popular among entomologists, but is necessary if we want to compare specimens from different euarthropod ingroups. Ingroup terms and more general crustacean terms are both given. This should also allow readers more specialised on other groups to follow while keeping the original reference.

## Description and Structural Interpretation of the Specimen

The single specimen of *Rhyniognatha hirsti* is not easily understood at first view. It demands for a more careful step-by-step interpretation of the preserved structures. In the following we are trying to provide such a clear stepwise interpretation of all observable structures. This will be done sub-structure by sub-structure; for each we will provide (1) the direct observation, (2) the former interpretation based on older imaging techniques, and (3) the new structural interpretation based on the new images. While this partly drags aspects of a classical discussion into a descriptive part, we think it still provides a clear distinction between observed and interpreted features while providing a very direct and comprehensible connection between these aspects. Further-reaching evolutionary interpretations will be provided separately in the discussion.

### General overview

**Figure 1:**
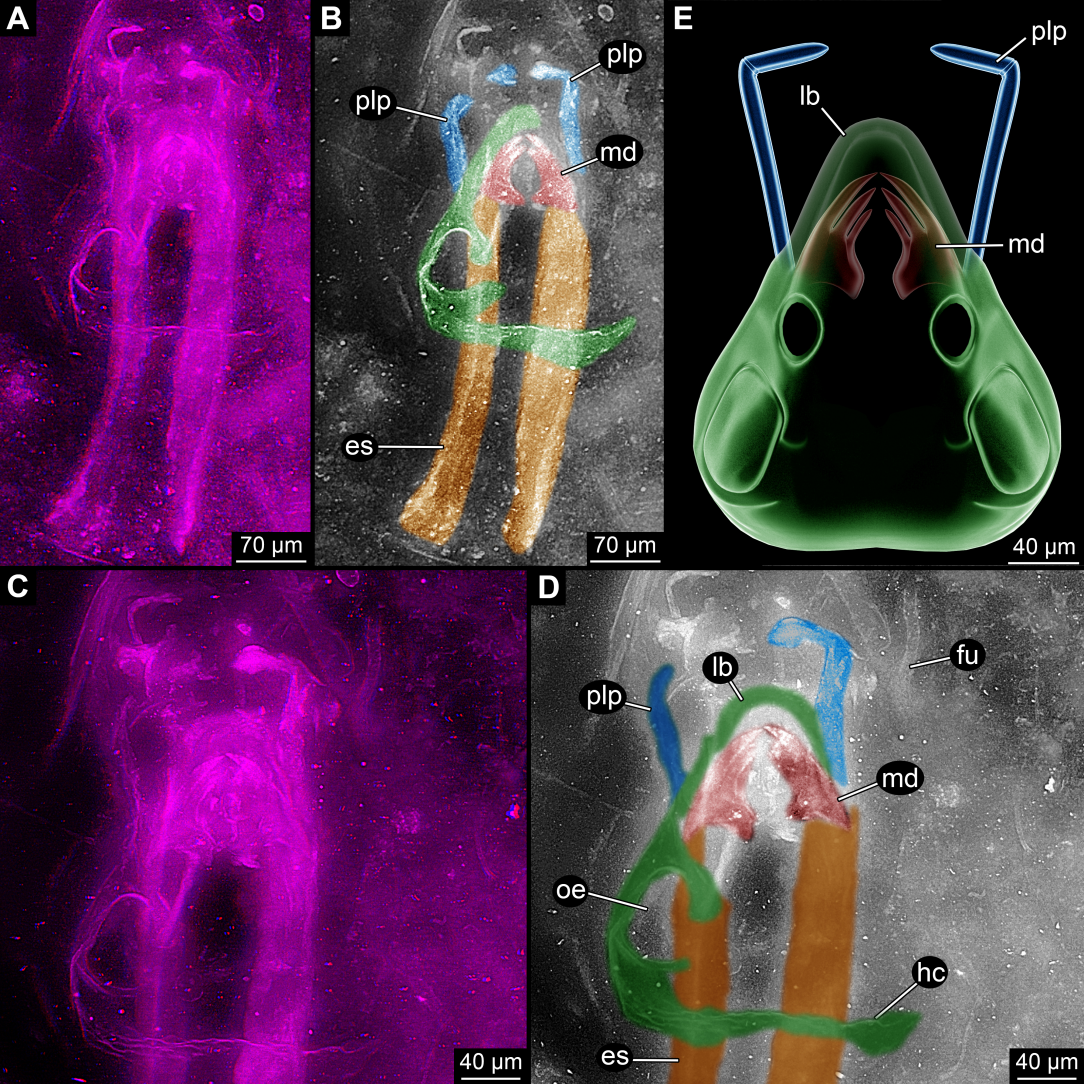
Overview images of single specimen of *Rhyniognatha hirsti*. (A) Overview of all structures, stereo-anaglyph. (B) Interpretation of (A). (C) Close-up on anterior structures, stereo-anaglyph. (D) Interpretation of (C). (E) Simplified restoration of observed outer structures. Abbreviations: es, elongate structures; fu, fungus; hc, head capsule; lb, labrum or clypeo-labral complex; md, mandibles; oe, possible opening for the compound eye; plp, palp.

**Figure 2:**
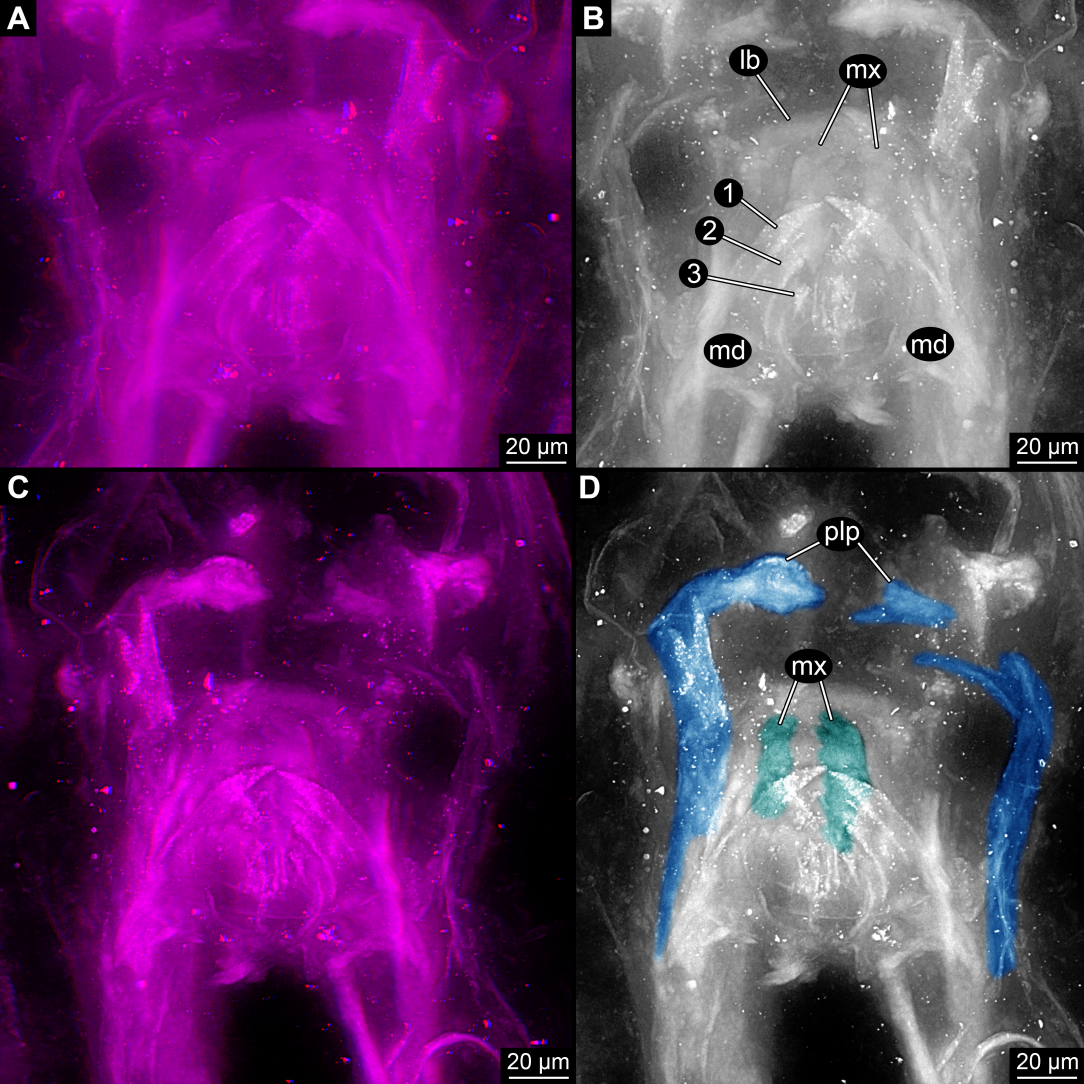
Close-up images on mouth parts of *Rhyniognatha hirsti*. (A) “Normal”, dorsal view, stereo-anaglyph. (B) Interpretation of (A). (C) Image flipped, quasi-ventral view, stereo-anaglyph. (D) Interpretation of (C) Abbreviations: 1–3, teeth-like protrusions of mandibles; lb, labrum or clypeo-labral complex; md, mandibles; mx, possible ‘first maxillae’ (= maxillulae); plp, palp.

The specimen consists of several more or less connected pieces of supposedly former cuticle:

(1) Most prominent is a pair of jaw-like structures (more details further below; Fig. 1). These jaw-like structures have been interpreted as mandibles (e.g., Engel & Grimaldi, 2004 and references therein; Grimaldi & Engel, 2005), which is supported by our observation.

(2) Topologically slightly below these mandibles is a pair of two elongate structures (Figs. 1A–1D). Their anterior ending is difficult to observe, but appears to be close to (or in?) the mandibles. They are about as wide as the mandibles and extend about eight times as long as the width towards the posterior. The principal shape is reminiscent of a shoe horn, i.e., roughly like a concave rail. The surface appears softer, less sclerotised, partly folded and warped. This structure has been supposed to represent either remnants of the head capsule (Grimaldi & Engel, 2005) or apodemes (Engel & Grimaldi, 2004). The head capsule interpretation appears unlikely under the new observations: (a) The paired nature of these structures is hard to understand in this interpretation. (b) The apparent softness of the structures is also not to be expected from a well-sclerotised structure as the head capsule. (c) The relative position in comparison to the mandibles makes this interpretation also unlikely; these structures appear to be slightly ventral to the mandibles (see also further below), unusually so for parts of the head capsule. Also, the almost in level position to the mandibles is unusual in this aspect. The softness and this position point to the possibility that these structures are indeed internal structures. A further reaching discussion is given further below.

(3) A pair of anterior protruding structures (Fig. 1). These appear to start from slightly below the mandibles, but reach the same level further anterior. These structures were interpreted as additional mouthparts, maxilla (Grimaldi & Engel, 2005) or more precisely the lacinia (Engel & Grimaldi, 2004). In both interpretations the interpretive drawings indicate the presence of more than one forked structure. Yet after close inspections of the new images in 3D (Fig. 2) it appears more likely that these supposedly independent structures are indeed continuous and represent a single, continuous structure. This would mean that the structure is very elongate and then suddenly bent inwards, suggesting the presence of a joint at this point. We therefore think that the structure more likely represents not the medial part of a mouthpart, but a distal one, a palp.

(4) The mandibles are partly “surrounded” by a cap-like structure (Fig. 1). Especially anterior to the mandibles this is apparent. The structure was recognised by Engel & Grimaldi (2004) and Grimaldi & Engel (2005) but not further interpreted. The authors drew the structure as heavily wrinkled. This wrinkling cannot be supported by our observation, the structure appears to be comparably smooth and sclerotised. Based on position and shape it most likely represents the labrum or clypeo-labral complex.

(5) Laterally and postero-laterally a larger structure is apparent (Fig. 1). It is hardly visible in earlier photographs as it largely lies in a focal plane above the mandibles. It was also not included in the interpretive drawings of Engel & Grimaldi (2004) and Grimaldi & Engel (2005). The structure is asymmetrical. Based on position and “texture” it is most likely part of the original specimen and more or less in its original relative position to the other parts. The right side is interpreted as damaged and incomplete. Posteriorly there is a larger part of cuticle; it is above the elongate paired structures and almost perpendicular to the angle of view. The surface appears wrinkled on first sight. Yet this seems not as being indicative of softness, but more appearing like a rougher and heavier sclerotised surface. The surface curves forward and also slightly inwards, coming close to the mandibles and continuing towards the possible labrum. Additionally, small “bars” extend from the main part dorso-medially. One surrounds a more or less circular opening comparably far posteriorly. A second weaker one is further anterior (seen in higher magnification). Based on the position this structure is most likely a partially preserved head capsule. The “bars” most likely represent sclerotised areas between unsclerotised ones, possibly indirectly indicating the original positions of the (compound) eye and antenna (=antennula in euarthropod terminology). The entire structure is most likely (the remain of) the head capsule.

(6) Anterior to the fossil appear to be more cuticle fragments (Figs. 1A and 1B). These do not appear to be connected to the other parts of the specimen. It remains unclear whether they have been originally part of the specimen or originate from another organism.

(7) Close to the fossil is the remain of a fungus (Figs. 1C and 1D).

### Smaller details

**Figure 3:**
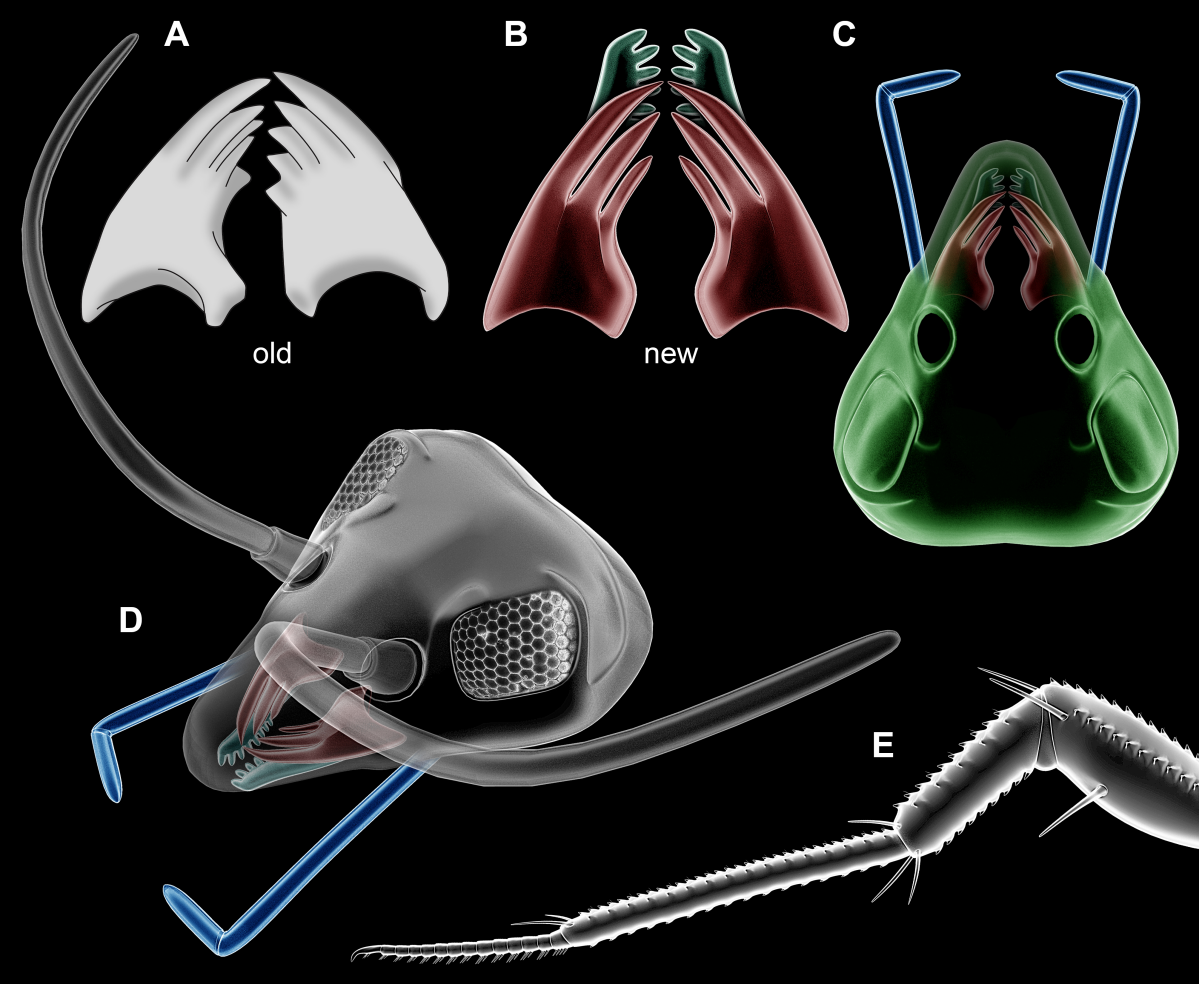
Partial restorations. (A–D) *Rhyniognatha hirsti*. (A) Old interpretation of mandibles, redrawn after Engel & Grimaldi (2004). (B) New interpretation of mandibles and possible maxillae in relative position. (C) Amended restoration (from Fig. 1E). (D) Further amended restoration, with supposed antennae (only outline, no subdivisions of flagellum) and compound eyes (from *Scutigera coleoptrata* photograph). (E) Leg of a representative of *Crussolum* (after Shear, Jeram & Selden, 1998).

(1) The mandibles have prominent functionally median tooth-like protrusions. We can directly observe three of these (Figs. 2A and 2B). Engel & Grimaldi (2004) and Grimaldi & Engel (2005) observed five of these (Fig. 3A). In their images the same three as observed by us (Fig. 3B) are very apparent. The other two are not clearly visible, they might possibly be structures of other mouthparts. Yet the mandible morphology only becomes apparent under very high magnifications and could probably not be identified clearly with older methods. A distinctly set off molar part is not apparent. Also the more proximal regions, where the supposed condyles are to be expected, is more difficult to interpret. While we cannot fully exclude the presence of a so-called ‘acetabulum’ (as described by Engel & Grimaldi, 2004), it does not show up as clearly as indicated by the interpretive drawings of Engel & Grimaldi (2004) due to preservation.

(2) Another pair of structures can be seen below the mandibles in higher magnifications (Figs. 2C and 2D). They are largely concealed by these. In a depth inverted image these structures can be seen above the mandibles and hence better understood; yet this way of presentation has the disadvantage that the fainter structures are in front of the better outlined mandibles which can be confusing on first view. The structures are paired and comb-like. Most likely these represent parts of additional mouthparts, possibly first maxillae (=maxillulae).

## Discussion

### Insect affinities

Engel & Grimaldi (2004) argued that *Rhynignatha hirsti* is an insect (“hexapod”) with certainty, most likely a representative of Pterygota. Other authors before have argued that *R. hirsti* might represent a myriapod (Rasnitsyn & Quicke, 2002). Our new observations do not heavily support that the characters of the mandibles argue for an insect ingroup position. Hence we will in the following compare the possible affinities not only to insects, but also to other possible arthropods.

### The mandibles

The mandibles have been interpreted to have an acetabulum, a major indicator for the insect interpretation. The specimen might have had an acetabulum and a differentiation into molar and incisivus. Yet these aspects cannot be clearly observed. The presence of these structures cannot be excluded but is also not positively supported, and therefore needs to be taken with care.

The mandibles (Fig. 3B) do not appear to have born palps, making an interpretation as an insect mandible likely, but also leaving possibilities for a myriapod interpretation or another type of crustacean with a palp-less mandible (compare e.g., Edgecombe, Richter & Wilson, 2003, their fig. 1). Massive teeth-like structures are known from insect mandibles, but also from those of myriapods. Based on the mandible morphology a clear decision between insect vs. myriapod cannot be made.

### The head capsule

As the mandibles are in fact less telling than assumed before, the other structures surrounding them become more important, especially the head capsule (Figs. 3C and 3D). The sclerotic bars indicate that this structure is indeed capsulate surrounding eyes and antennae (=antennulae in euarthropod terminology). While capsulate heads are known also in isopods, these do not surround the antennula (corresponding appendage in crustaceans). The structure seen in *R. hirsti* is therefore indicative of a myriapod or insect type head capsule.

Insect head capsules are very confined posteriorly, providing a high movability. The head capsules of myriapods are still more shield-like, especially in centipedes, in some cases strongly resembling the overall arrangement of *R. hirsti* (compare to Edgecombe, 2006, his fig. 1). Also the condition with a wider, less confined posterior opening seems be present in *R. hirsti* (see also next point) arguing more for a myriapod interpretation.

### The elongate structures

A centipede-like arrangement of the head capsule is supported by the arrangement of the elongate structures. These arise from close to the base of the mandibles and protrude far posteriorly beyond the limitations of the head capsule. As the insect-type head capsule is so strongly confined posteriorly (forming a narrow foramen) no larger internal structures can extend into the trunk (besides, of course, some muscles, nervous system and the gut). The elongate structures could represent muscles or apodemes (as suggested by Engel & Grimaldi, 2004); yet in chilopodan myriapods (centipedes) there are also glands of ectodermal origin ending in the mandibles that are in pleurostigmophoran centipedes protruding far into the trunk (e.g., Koch et al., 2011, their fig. 6.1). Hence the elongate structures may represent mandibular glands. This could explain their cuticular appearance (ectodermal invaginations) while being soft-appearing. According to Dunlop et al. (2004), certain parts of the Rhynie chert can preserve soft-tissue such as muscles, digestive or reproductive organs. If the interpretation as mandibular glands is accepted, it further supports an interpretation of *R. hirsti* as a myriapod.

### Other mouth parts

**Figure 4:**
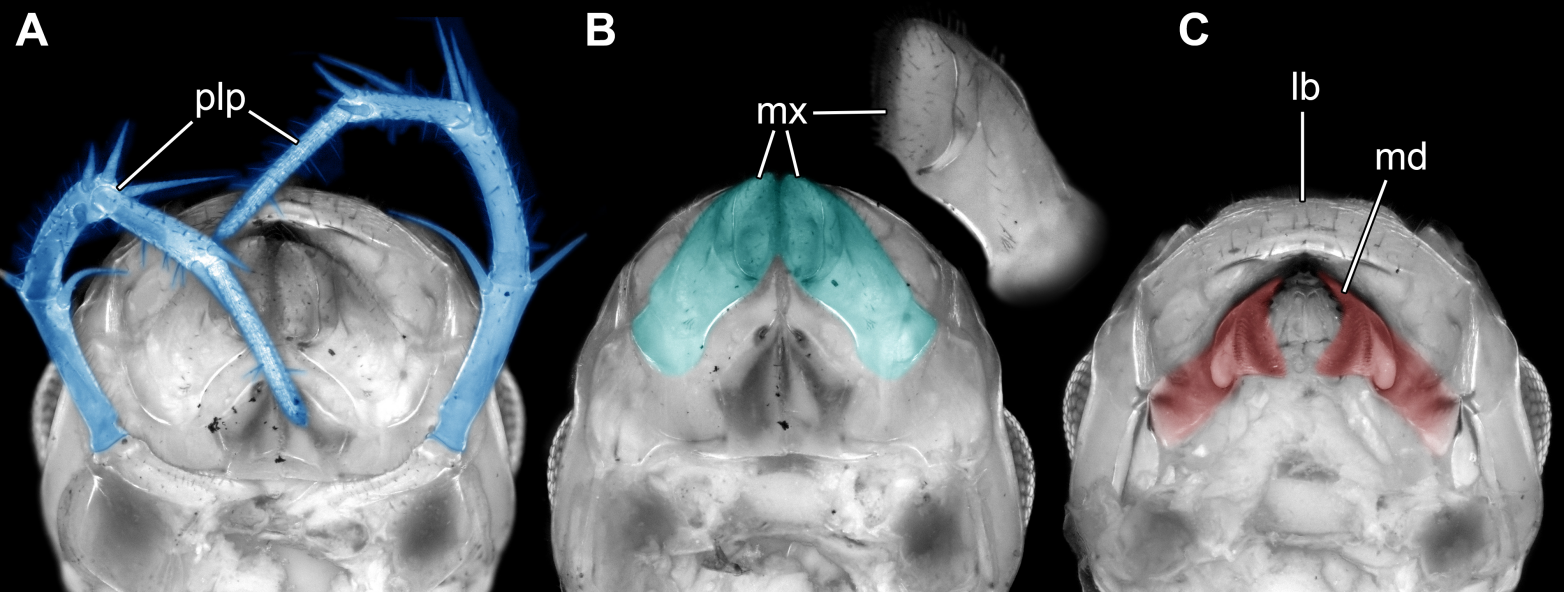
Head of the extant scutigeromorph *Scutigera coleoptrata* in ventral view, composite autofluorescence image. (A) With ‘second maxilla’ (= maxilla) still attached. (B) Maxilla removed revealing the ‘first maxilla’ (= maxillula). (C) Maxillula removed revealing mandible. Abbreviations: lb, labrum or clypeo-labral complex; md, mandibles; mx, possible ‘first maxillae’ (= maxillulae); plp, palp.

The comb-like structures could represent part of a chilopod-like ‘first maxilla’ (maxillula; Figs. 3B and 3D), distantly resembling those of extant forms (e.g., Bonato et al., 2010; Minelli, 2011, his fig. 3.4). In insects such comb-like structures would be more unusual. If interpreted as a chilopod, the elongate palp would then most likely represent the palp of a (second) maxilla (Fig. 4; e.g., Bonato et al., 2010; Minelli, 2011, his fig. 3.4); however also an insect type labial palp would be compatible. Still the additional mouth parts seem to support a myriapod interpretation.

**Figure 5:**
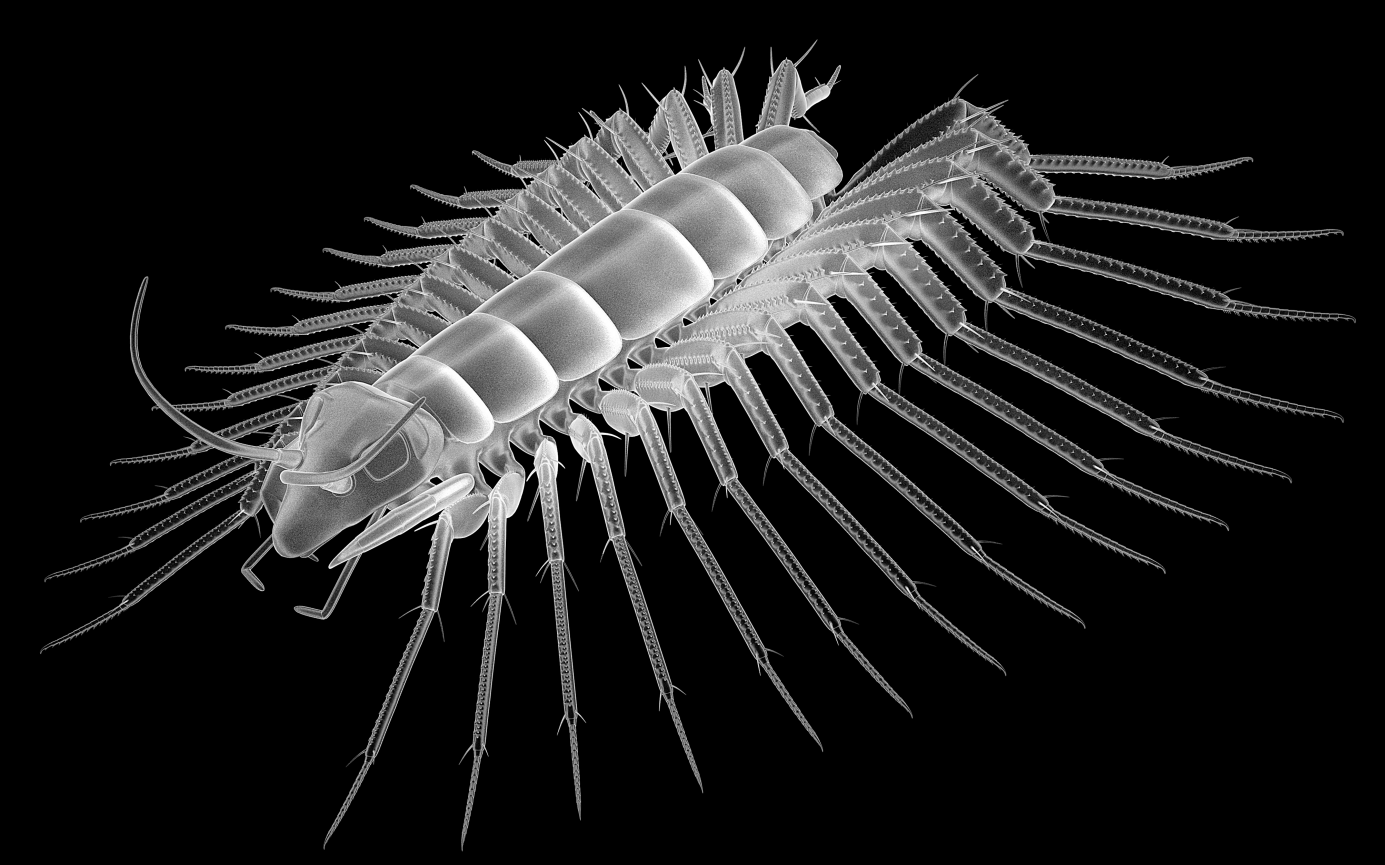
Newly suggested, highly speculative interpretation of *Rhyniognatha hirsti* as a *Crussolum*-like centipede. Note that the depicted morphology refers to a fully grown adult, while the fossil itself was more likely an early immature.

In summary we cannot fully exclude an insect affinity of *R. hirsti*, as the specimen is very incomplete and the supposed key characters of the mandible are at best difficult to observe. Yet given the observable characters of the structures surrounding the mandible, a myriapod interpretation is in our view better supported.

These details would be best compatible with an interpretation as an early centipede (Chilopoda). Centipede fragments from Rhynie chert (in the wide sense) include isolated legs and a venom claw; these have been named *Crussolum* (Fig. 3E) and been interpreted as an early representative of Scutigeromorpha (Shear, Jeram & Selden, 1998). Such organisms are quite large, in the centimeter range, also fossil forms known from the Carboniferous (see e.g., Haug et al., 2014). Yet early stages of scutigeromorphs are rather small in size. We therefore speculate that the single specimen of *R. hirsti* is an early post-embryonic stage of a myriapod (Fig. 5). It must remain unclear if the fragments already known from *Crussolum* are conspecific with *R. hirsti*, but the observed structures at least give a stronger support for an interpretation as a myriapod than for an interpretation as an insect.

The discussion presented here appears to follow a common scheme: Differentiating a fossil myriapod from an early insect is not trivial. Not only are the cases of *R. hirsti* or *Leverhulmia mariae* (Anderson & Trewin, 2003; Fayers & Trewin, 2005) comparable, but to a certain extent younger fossils have also been discussed in a similar way (e.g., Kukalová-Peck, 1997; Haug et al., 2015 vs. Willmann, 1997; Grimaldi & Engel, 2005; Nel et al., 2007). Another problem which needs to be mentioned in this context is a certain tendency to overinterpret exceptional fossils (see, for example, discussions in Donoghue & Purnell, 2009; Klug et al., 2015; Sallan et al., 2017).

The insect vs. myriapod distinction in early fossils will most likely provide more discussions in the future. The early fossil records of insects still remains scarce and problematic, and are now possibly scarcer than before.

## Additional Information and Declarations

### Data Availability

The following information was supplied regarding data availability: The images are deposited in MorphDBase (ID numbers: C_Haug_20170507-M-10.1 to C_Haug_20170507-M-16.1):
http://www.morphdbase.de/?C_Haug_20170507-M-10.1

http://www.morphdbase.de/?C_Haug_20170507-M-11.1

http://www.morphdbase.de/?C_Haug_20170507-M-12.1

http://www.morphdbase.de/?C_Haug_20170507-M-13.1

http://www.morphdbase.de/?C_Haug_20170507-M-14.1

http://www.morphdbase.de/?C_Haug_20170507-M-15.1

http://www.morphdbase.de/?C_Haug_20170507-M-16.1.
